# Book Reviews

**Published:** 1897-02

**Authors:** 


### *BOOK REVIEWS.*

---

The Principles of Theoretical Chemistry, with Special Reference to the Constitution of Chemical Compounds. By Ira Remsen, Professor of Chemistry in the Johns Hopkins University. Fifth edition, thoroughly revised. Lea Brothers & Co., Philadelphia and New York, 1897.

To the student of chemistry there is nothing so essential to his comprehensive grasp of the science as an intelligent appreciation of its fundamental principles, and that writer accomplishes the most in aiding him to learn who states these principles in a manner which enables the mind to easily grasp them.

The first seven chapters of the book are devoted to an exposition of the Laws of Combination. Beginning with the Law of Definite Proportions, in turn are explained the Investigations of Dalton, and the promulgation of his Theory, and the various methods for determining the Atomic Weights of the Elements. The seventh chapter is devoted to a consideration of Valency; this property is one that seems particularly difficult of comprehension by the student, and so clear and concise a description of it will aid materially in its being understood. The remainder of the volume is devoted to the study of the Constitution and Structure of Chemical Compounds, taking in turn the inorganic or those not containing carbon; and the organic, or those containing carbon; the monobasic acids; and the benzene derivations.

Chapter seventeen treats of the Physical Methods used for determining the constitution of chemical compounds.

Chapter eighteen is given up to a study of Chemical Affinity, while the concluding chapter deals with the relations between the chemical constitution and the properties of compounds.

This work will be a great aid to every student of chemistry, and will throw much light upon various portions of the science which have hitherto been either completely obscured from his mental vision or at least very hazy. M. A. P.

ESSENTIALS OF PHYSICAL DIAGNOSIS OF THE THORAX. By Arthur M. Corwin, A.M., M.D., Demonstrator of Physical Diagnosis in Rush Medical College, etc. Second edition, revised and enlarged. W. B. Saunders, Philadelphia, 1896. Price $1.25.

The necessity for a second edition of this work shows the need of clear and concise presentation of medical facts for students; and further that when such a work appears it is appreciated. It is essentially a work for the class-room, calling attention to cardinal principles, and leaving for further study the more elaborate theories of the subject; this fixes facts upon the mind, without clogging the memory with burdensome speculations.

The arrangement of the subject-matter is good, and when used to follow the demonstrator of physical diagnosis, it will be of invaluable benefit to the student.

The divisions of the work include: (1) Topography of the Chest; (2) Landmarks of the Chest; (3) Methods of Physical Diagnosis; (4) Physical Signs Common in and Peculiar to each Disease of the Chest.

The author has wisely given some space to a consideration of the *Pulse*, a subject often slurred over in many works otherwise good. A careful perusal of this portion will be of advantage to practitioner and student alike. M. A. P.

---

THE PRACTICE OF MEDICINE. A Text-book for Practitioners and Students with Special Reference to Diagnosis and Treatment. By James Tyson, M.D., Professor of Clinical Medicine in the University of Pennsylvania and Physician to the Hospital of the University; Physician to the Philadelphia Hospital; Fellow of the College of Physicians of Philadelphia; Member of the Association of American Physicians, etc. In one volume, pp. 1,174; illustrated. Price $5.50. P. Blakiston, Son & Co., 1012 Walnut street, Philadelphia.

This book is the result of several years' labor. "It has taken some time, because it represents almost purely personal work, which has been frequently interrupted. It does not pretend to be based on my personal practice alone." It is a work of the size and nature suitable for the student, but is sufficiently comprehensive to be of value also as a reference work for the practitioner, though it cannot compare with modern medical encyclopedias for the latter purpose. Although it was not at first the author's intention to illustrate the text, he has found it necessary to produce about 100 charts and other drawings, mostly of a somewhat unpretentious nature.

The author first defines the disease under consideration, then gives a succinct sketch of its history, before proceeding with the etiology, morbid anatomy, symptoms and treatment. Each of these receives what seems to be a very proper share of his attention, according to its importance. We have observed nothing to which exception can be taken, and the book we consider to be one which can be fairly recommended to those in need of such a work. A. W. S.

---

AUTOSCOPY OF THE LARYNX AND THE TRACHEA. By Alfred Kirstein, M.D. Authorized translation by Max Thorner, A.M., M.D., Cincinnati, O., Professor of Clinical Laryngology and Otology, Cincinnati College of Medicine and Surgery ; Laryngologist and Aurist, Cincinnati Hospital, etc. Twelve illustrations. Extra cloth, 75 cents, net. The F. A. Davis Co., Publishers, 1914 and 1916 Cherry street, Philadelphia.

The author here describes the new method of direct examination of the larynx and the traehea without the reflecting mirrors in common use. The Autoscope consists essentially of a well illuminated (electricity) tube mounted on a long spatula, which depresses and controls the tongue. With this instrument, and the patient's head in proper position, direct inspection of the larynx may be made and a true picture of the parts obtained, instead of a reflected and reversed image. The author describes the instrument and the technique of its application. The translator considers the "autoscopic examination of the air passages as developed by Kirstein the most important addition to our technical resources made since the discovery of the laryngoscope by Garcia." We believe that this instrument has a great future. The chief obstacle to its use will be the intolerance of most throats.

We cannot but object to the name given to this method. This is not *autoscopy* at all, and it is no more proper to apply this term to direct inspection of the larynx than to a similar direct examination of the middle ear or of the interior of the bladder. It is, however, *laryngoscopy* by a direct method and the apparatus is a true laryngoscope.

---

THE JOURNAL OF CUTANEOUS AND GENITO-URINARY DISEASES.

With the issue of January, 1897, Number 1, Vol. XV, this valuable journal appears with a new editorial staff and new publishers. Dr. Jas. C. Johnston and Dr. Geo. K. Swinburne are the editors, Dr. Johnston appearing as acting editor. The collaborators are Louis A. Duhring, M.D., John A. Fordyce, M.D. (formerly editor), Edward L. Keyes, M.D., Prince A. Morrow, M.D., Robt. W. Taylor, M.D., and Jas. C. White, M.D The editors are bright and able men in their field and the names of the collaborators afford a sufficient guarantee that the standard of the journal will be of the highest. This is the organ of the Dermatologists, Syphilographers and Genito-Urinary specialists in America. The best in these branches is published there, and it is the journal of reference for those interested in these subjects.

The scope of the *Journal* will be enlarged to admit more from other periodicals, and editorials will be added.

We hope the cover of the next issue will be in the old distinctive color, and that the new editors will demand extra vigilance upon the part of the proof-readers with a view to the avoidance of typographical errors, which so often mar medical papers. We wish the *Journal* increased success, and advise all who are interested in the subjects treated in its pages to become its active supporters.

---

INTERNATIONAL MEDICAL ANNUAL.

E. B. Treat, publisher, New York, has in press for issuance early in 1897, the *International Medical Annual*, being the fifteenth yearly issue of that well-known one-volume reference work. The prospectus shows that the volume will be the result of labors of upwards of forty physicians and surgeons, of international reputation, and will present the world's progress in medical science.

The volume will contain about 700 pages. The price will be the same as heretofore, $2.75.

